# Astrocyte-Secreted Factors Modulate a Gradient of Primary Dendritic Arbors in Nucleus Laminaris of the Avian Auditory Brainstem

**DOI:** 10.1371/journal.pone.0027383

**Published:** 2011-11-07

**Authors:** Matthew J. Korn, Scott J. Koppel, Karina S. Cramer

**Affiliations:** Department of Neurobiology and Behavior, University of California Irvine, Irvine, California, United States of America; University of Southern California. United States of America

## Abstract

Neurons in nucleus laminaris (NL) receive binaural, tonotopically matched input from nucleus magnocelluaris (NM) onto bitufted dendrites that display a gradient of dendritic arbor size. These features improve computation of interaural time differences, which are used to determine the locations of sound sources. The dendritic gradient emerges following a period of significant reorganization at embryonic day 15 (E15), which coincides with the emergence of astrocytes that express glial fibrillary acidic protein (GFAP) in the auditory brainstem. The major changes include a loss of total dendritic length, a systematic loss of primary dendrites along the tonotopic axis, and lengthening of primary dendrites on caudolateral NL neurons. Here we have tested whether astrocyte-derived molecules contribute to these changes in dendritic morphology. We used an organotypic brainstem slice preparation to perform repeated imaging of individual dye-filled NL neurons to determine the effects of astrocyte-conditioned medium (ACM) on dendritic morphology. We found that treatment with ACM induced a decrease in the number of primary dendrites in a tonotopically graded manner similar to that observed during normal development. Our data introduce a new interaction between astrocytes and neurons in the auditory brainstem and suggest that these astrocytes influence multiple aspects of auditory brainstem maturation.

## Introduction

The convergence of binaural information in the central auditory nervous system is necessary for sound source localization. The avian auditory brainstem contains a well-characterized circuit with several anatomical and physiological features that facilitate the encoding of temporally sensitive information [1], [2], [3], [4]. Auditory VIIIth nerve afferents send tonotopically organized information to neurons located in the ipsilateral nucleus magnocellularis (NM), which in turn project bilaterally to make contact on bitufted dendrites and cell bodies in nucleus laminaris (NL) [5], [6], [7]. Axons from NM bifurcate and the ipsilateral branch terminates along dorsal NL dendrites, while the contralateral branch forms delay lines that terminate along ventral dendrites in NL. Tonotopic information from NM is preserved in NL, such that relatively high frequency sounds are processed in neurons in the rostromedial portion of the nucleus while lower frequencies are processed towards the caudolateral pole [8], [9]. Maximal excitation of a single NL neuron occurs when segregated input from both ears arrives simultaneously [10]. NL neurons thus act as coincidence detectors and compute interaural time difference (ITD), which encodes the location of sound in space [11], [12], [13].

Neurons in NL exhibit a gradient of dendritic arbor size that varies systematically along the tonotopic axis [14]. Starting at embryonic day 15 (E15) several features become apparent that contribute to the perceived gradient along the tonotopic axis [14], [15]. The rostromedial (high frequency) neurons become shorter and as a result, their total dendritic length is 13-fold smaller than caudolateral (low frequency) neurons [15]. There is a loss in the number of primary dendrites in the caudolateral region of NL from E15 to E19 that results in a 30-fold gradient in the number of primary dendrites, so that low frequency neurons have far fewer primary dendrites than high frequency neurons [15]. These primary dendrites are also longer in the caudolateral-third compared to the rest of the NL cell layer [14]. Dendrite extension, or the distance of the furthest terminal from the soma, also varies systematically along the frequency axis, with neurons demonstrating the longest extension in the caudolateral region of NL [14].

It was initially hypothesized that changes to dendritic morphology come about after hearing onset when afferent activity is known to provide trophic support and promote growth [15], [16]. Parks and colleagues tested this hypothesis by preventing the formation of the inner ear and VIIIth nerve afferents, only to find that while the total length of dendrites did decrease, the overall structure of the dendritic gradient remained intact [17], [18]. Studies on changes to NL neurons after deafferentation report immediate and persistent dendritic atrophy followed by resorption of the deafferented dendrites into the soma [19], [20], [21], [22], [23]. Though activity-dependent mechanisms are necessary for proper development and maintenance of dendrites in the auditory brainstem [24], [25], no experimental perturbation has previously resulted in the loss of the characteristic gradient of dendritic morphology.

This period of dendritic reorganization in NL, beginning at about E15, coincides with the emergence of astrocytes that express glial fibrillary acidic protein (GFAP) [26]. Glial cell bodies are restricted to the margins outside the NL neuropil during embryonic development [27], [28], [29], [30], [31], and GFAP-positive astrocytes extend processes within the dendritic neuropil at the age when dendritic arborization patterns are changing [26]. Several studies have provided evidence to support a role for Bergmann glia in establishing the dendritic arbors of Purkinje cells in the development of the cerebellum [32], [33]. Evidence from *C. elegans* suggests that glia are also necessary for modulating the shape of developing dendritic arbors [34]. Interactions between neurons and glia *in vitro* indicate that glia are necessary for development of neuron morphology and dendritic arbor support [34], [35]. However, relatively little is known about the role of glia in the auditory system, and the contribution of glia to branch order in dendritic morphology has not been explored. We used a heterchronic organotypic slice preparation in which astrocyte-derived factors from older embryos were presented to labeled NL neurons in slices taken prior to maturation of GFAP-positive astrocytes. We then used morphometric analysis of live images to test whether astrocyte-derived proteins contribute to the emergence of dendritic features that are graded along the tonotopic axis. Our results suggest that factors secreted from brainstem astrocytes promote the formation of the gradient of primary dendrites.

## Materials and Methods

### Tissue

Brainstem slices and astrocytes were obtained from fertilized white leghorn eggs (AA Laboratories), which were set in a rotating incubator at 39°C and used at ages E13, E16 and E17.

### Normative Measurements

Our strategy for this study relies on organotypic brainstem slices from coronal sections. Because the tonotopic position is difficult to determine accurately in this preparation, we first made normative measurements of neurons labeled in acute preparations cut along the tonotopic axis to identify NL dendrite features that reliably correlate with tonotopic position. We cut sections of E13 and E17 brainstems at an angle 30° to the sagittal plane in cold oxygenated ACSF at 200 *µ*m [36] on a VT1000P Vibratome (Leica Microsystems, St. Louis, MO). In this orientation, one to two sections containing all or most of the entire extent of the tonotopic axis [8], [14] were collected and neurons were filled iontophoretically as described below. In addition, to obtain detailed information on changes that take place in NL dendrites, we studied neurons filled in acutely cultured coronal sections at E13 and E17.

### Dissociated Astrocyte Culture and Collection of Conditioned Medium

Astrocyte isolation was adapted from previous studies [37], [38]. Briefly, E16 brainstems were trypsinized (0.05% trypsin-EDTA) and the tissue was dissociated in DMEM/F12 containing penicillin/streptomycin (astrocyte base medium, ABM) with 10% fetal bovine serum (astrocyte growth medium; AGM) and 100 *µ*l of bovine serum albumin (BSA). The resulting supernatant was layered above AGM containing 4% BSA and centrifuged. The pellet containing the dissociated cells was resuspended in AGM, poured through a 40-*µ*m cell strainer, and transferred to a T-75 flask pre-treated with poly-d-lysine (50 *µ*g/ml; Sigma-Aldrich, St. Louis, MO). Medium was replaced every 48 hours until cultures were confluent.

To produce fibrous astrocytes, confluent flasks were treated with 1 *µ*M cytosine arabinoside in ABM for 24 hour. The cells were then replated in ABM containing G5 supplement (1∶100; 500 g/ml insulin, 5 mg/ml human transferrin, 0.52 g/ml selenite, 1 g/ml biotin, 0.36 g/ml hydrocortisone, 0.52 g/ml FGF_2_ and 1 g/ml EGF, ADM; Invitrogen, Carlsbad, CA) at 3×10^6^ cells per T-75 flask. Once confluent, the cultures were exchanged to ABM (without G5) to collect astrocyte-secreted factors for 3–4 days. The astrocyte-conditioned medium (ACM) was concentrated using an Amicon Ultra 10K centrifugal filter (Millipore, County Cork, Ireland) and assayed for protein concentration. The ACM used in our experimental treatment group ranged between 25–33 *µ*g/ml. ACM used for low dose treatments had a concentration 15–20 *µ*g/ml.

### Organotypic Slice Preparation

We prepared organotypic brainstem slices according to previously published studies with modifications to allow for multiday imaging [39], [40]. Physiological properties of chick auditory brainstem neurons have been previously shown to be preserved *in vitro* [41]. Embryos were removed at E13 and dissected so as to keep the brainstem together with cochlea, cochlear ganglion, and VIIIth nerve intact. We included these peripheral structures and innervation to prevent effects of deafferentation on the number of NM neurons and the subsequent atrophy of NL dendrites [23], [42], [43], [44]. The tissue was immediately placed into cold culture medium containing 50% advanced minimum essential medium (MEM; Invitrogen, Carlsbad, CA), 25% Earle's balanced salt solution (EBSS; Sigma-Aldrich, St. Louis, MO), 25% normal horse serum (NHS, heat-inactivated and filter sterilized; Invitrogen, Carlsbad, CA), supplemented with 200 mM L-glutamine (Sigma-Aldrich, St. Louis, MO), D-glucose (5.5 mg/ml; Sigma-Aldrich, St. Louis, MO), penicillin and streptomycin (10,000 unit/ml, 10,000 *µ*g/ml; Invitrogen, Carlsbad, CA). After a brief rinse, tissue was placed in 4% low-melting point agarose in artificial cerebrospinal fluid (ACSF; 130 mM NaCl, 3 mM KCl, 1.2 mM KH_2_PO_4_, 20 mM NaHCO_3_, 3 mM HEPES, 10 mM Glucose, 2 mM CaCl_2_, 1.3 mM MgSO_4_) perfused with 95% O_2_ and 5% CO_2_, and sectioned at 400 *µ*m on a Vibratome.

Each brainstem yielded two to three sections roughly corresponding to the rostral, middle or caudal region of the auditory brainstem. Slices were then transferred to a 0.4 *µ*m membrane Millicell-CM culture plate insert (Millipore, County Cork, Ireland) pre-treated with 200 *µ*l of a 0.01% polyornithine solution (Sigma-Aldrich, St. Louis, MO). Each membrane insert contained two slices and the insert was placed in a 3.46 cm diameter well with 1 ml of control culture medium (as described above). For proper adhesion to membrane inserts, these slices required equilibration for 24 hrs prior to the first medium exchange. Six-well trays containing inserts were maintained at 37°C and 80% humidity in a NAPCO Series 8000WJ water jacketed incubator (Thermo Electron Corporation, Marietta, OH) with 5% CO_2._ The following day, we performed *in vitro* labeling in NL neurons (see below), initial images were obtained, and wells were selected at random to receive a fresh exchange of either control medium or control medium supplemented with concentrated ACM (mixed 1∶1). We report the results after a single 24-hour period of treatment with ACM supplemented medium or control medium.

### *In vitro* labeling

To visualize NL dendritic arbors we labeled individual NL neurons with rhodamine dextran amine (RDA; MW  = 3000; Molecular Probes; 8–10% in sterile saline) as reported previously [23], [25], [38]. Acute slices cut coronally or along the tonotopic axis were transferred to a charged Sylgard-coated platform and a dye filled electrode (10–35 V, 50 ms duration, train of 3–5 pulses) controlled by a BTX Electro Square Porator ECM 830 (Harvard Apparatus) was used to fill 6–8 neurons per slice. Neurons and their processes filled almost instantaneously and retained the dye through completion. Sections were immediately transferred to 4% paraformaldehyde (PFA) in 0.1 M phosphate buffer (PB) prior to being imaged.

For organotypic slices, membrane inserts containing cultured brainstem slices were removed from incubator, transferred directly to the Sylgard-coated platform, and covered with 1–2 ml of warm ACSF. In contrast to acute sections, 3–5 neurons were filled on either side of each coronal organotypic slice. Membrane inserts were returned to the incubator for 4–6 hours prior to being imaged.

### Imaging and Analysis

#### Two-photon Microscopy

Membrane inserts were removed from the incubator and affixed to a dish containing low melting agarose (Fisher Scientific, Fair Lawn, NJ). For immersion microscopy, the inserts were filled with culture medium heated to 37°C. Once the slice was in focus, the cell body layer was identified using only transmitted light.

To collect digital images of NL neurons and their dendritic arbors, we used a custom-made video-rate two-photon laser scanning microscope, as described previously [38], [45], [46], [47]. Briefly, excitation was provided by trains of 100 fs pulses at 750–800 nm from a Chameleon Ti:sapphire laser (Coherent, Santa Clara, CA). The laser beam was scanned at 30 frames per second and focused through a 20x water-immersion objective (numerical aperture 0.95; Olympus BX51WIF, Olympus America, Inc., Center Valley, PA). Emitted fluorescence light was detected by photomultipliers (Hamamatsu, Middlesex, NJ) to derive a video signal that was captured and analyzed using SlideBook 5.0 (Intelligent Imaging Innovations, Inc., Santa Monica, CA). Neurons located in the caudal third of NL were imaged at 0.5 pixels per µm and all others at 0.25 pixels per *µ*m. Stacks containing 50–150 images were acquired at 1µm per plane.

For fixed acute sections, neurons were excluded if any of the visible RDA labeling was discontinuous within a neuron or if the depth of the filled neuron impeded the identification of individual processes. In the case of multiday imaging, previously imaged neurons were identified on the second day by their mediolateral location within the slice relative to the beginning of the cell layer. Though several changes were apparent, neurons maintained their orientation within NL. Neurons were not imaged if there was overlap with nearby filled neurons that obstructed determination of individual neurons. After treatment, neurons were not reimaged if their processes had become discontinuous, if the cell body was irregular, or if RDA scatter from adjacent neurons had obscured the full dendritic tree. Each stack was filtered using a Gaussian distribution (sigma = 1.33) to reduce noise, and exported as individual TIFF files for analysis.

#### Image Analysis

Prior to analysis, neurons were reconstructed using Neurolucida software with the AutoNeuron module (Version 9.13, MicroBrightField (MBF) Bioscience, Williston, VT). Individual TIFF images were realigned to account for process drift and ensure accurate measurements [48]. The thickest process emanating from the soma was measured so as to define all larger fields as the cell body. All processes were traced and originated from within the contours of the soma as defined by AutoNeuron. Axons were easily identifiable as they could be traced over 10 planes and usually traveled outside the region of interest without any observable branching. Dendritic outgrowths from the cell body were defined as having diameters between 0.8 and 5.0 *µ*m and extended for a minimum length of 1.5 *µ*m away from the cell body. Branch nodes were placed where new prominences arose from a single branch or where two separate processes could be distinguished and extended for more than a 1.0 *µ*m. Since NL neurons generally lack spines [14], separate processes were considered to be dendritic branches. All tracing was performed blind to age or treatment. Neurons were traced by one of three individuals and accuracy was verified by an independent judge who confirmed the length and position of each branch through each image of the stack. Completed reconstructions were exported to Neuroexplorer software (MBF, Williston, VT). We performed a morphometric tree analysis that provides the total and average length of each branch while categorizing each into the appropriate dendritic branch order. From these data we obtained total dendritic branch length, total number of dendritic branches, average length of dendritic branches; as well as the number and length of primary, secondary, and higher branches. A Sholl analysis was performed using 10 *µ*m concentric circles to determine dendritic extension, and here we report the most distal intersection as the furthest terminal from the cell body.

Representative images were exported as TIFF files and edited using Jasc Photo Shop Pro 8 (Corel, Ottawa, Ontario, Canada). In some instances brightness and contrast were adjusted to reduce background labeling. Images were converted to grayscale, resized to 300 dpi, and cropped to fit the dimensions of each figure. Skeletonized 3D reconstructions were exported from Neurolucida Explorer with high quality trees.

### Statistics

All statistical evaluations were made using JMP 9 software (SAS, Cary, NC). To categorize neurons according to position along the tonotopic axis, we represent measurements along an axis of a continuous x-variable; position or total dendritic length. Residuals were plotted to check for normal distribution of values. Comparisons were made using a fit-model ANOVA by which the least-squared means of the slopes could be compared. Values associated with each slope are reported as follows: slope, correlation coefficient, and *P* value of the coefficient. Where appropriate values are reported as mean ± SEM. Individual mean comparisons were made using Tukey's HSD test.

## Results

### Normal Dendritic Arbors at E13 and E17

Before we could ascertain the effects of astrocytes on morphometric features of NL dendrites, we first examined the changes that take place in NL dendrites during normal development at the time of astrocyte maturation. These studies in part validate our analysis methods, as they permit comparison with prior studies that used the Golgi-Cox method to visualize neurons [14], [15]. Additionally, they expand on the previous studies and include measurements of additional morphological features, focusing on the early stage of dendritic reorganization.

#### Extrapolating Tonotopic Position

We first obtained morphometric data from NL dendrites from untreated E13 and E17 brainstems sectioned obliquely along the tonotopic axis to determine features that are best correlated with tonotopic location within NL. The tonotopic location of a neuron is difficult to determine in the coronal plane because of variations in sectioning, and due to flattening once maintained in culture for several days [49]. The initial data set was obtained from untreated, uncultured brainstem cut along an angle so that individual sections included the rostromedial to caudolateral extent of NL [8], [20], [36].

**Figure 1:**
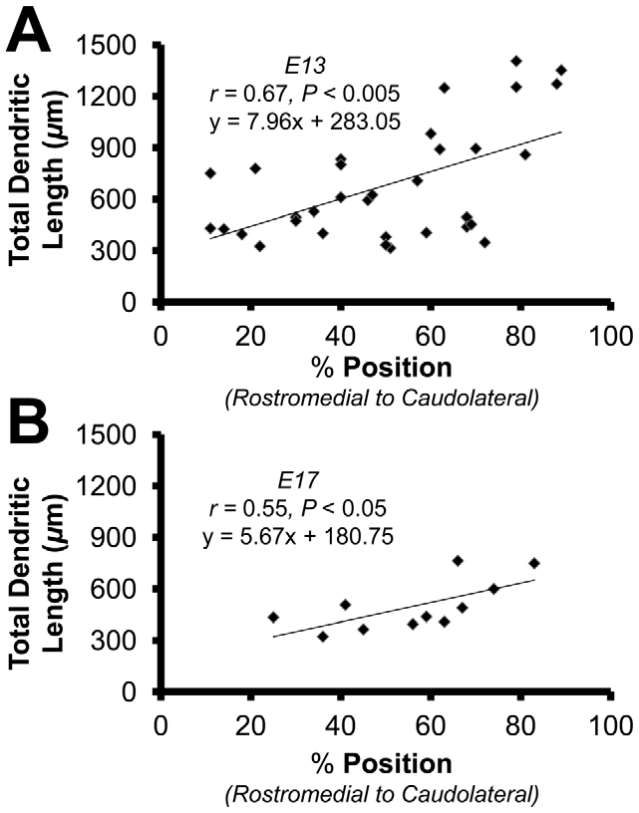
Total dendritic length is correlated with a neurons position along the tonotopic axis. **A**: Scatter plot, least-squares regression, and correlation coefficient for neurons sampled along the tonotopic axis at E13 (*n* = 33). **B**: Scatter plot, least-squares regression, and correlation coefficient for neurons sampled along the tonotopic axis at E17 (*n* = 11).

We found that the morphometric feature of dendrites that best correlated with tonotopic position at E13 was the total dendritic length, or the length of all the branch segments combined. We expressed position as a percentage along the axis from rostromedial (0%) to caudolateral (100%) (*n* = 33). The relationship between position and the total dendritic length was significant (slope  =  + 7.96, r = 0.67, *P*<0.005; Fig. 1A). This gradient was also observable at E17 (*n* = 11) and although the slope of the gradient (slope  =  + 5.67, r = 0.55, *P*<0.05; Fig. 1B) was not significantly different from E13 (*P* = 0.65*)*, there was a marked decrease in total dendritic length compared to E13 (*P*<0.05). Since the location of NL neurons can be reasonably approximated using total dendritic length at both E13 and E17, we used this measurement as an estimate of tonotopic position for all other comparisons when filled neurons from coronal sections included in the analysis.

#### Number of Primary Dendrites

**Figure 2:**
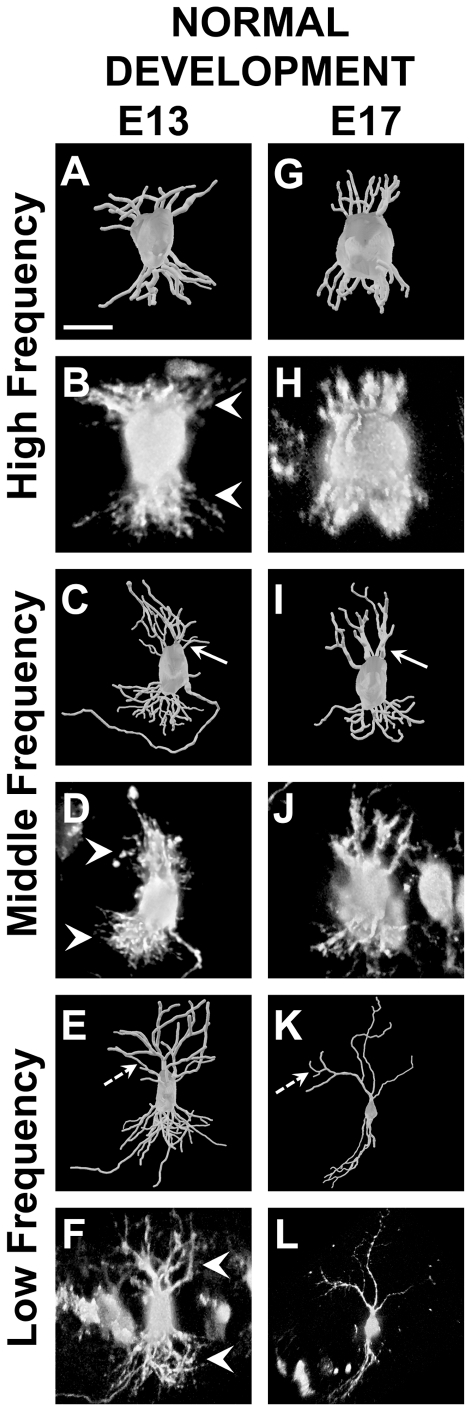
3D reconstructions and raw images of neurons from NL at E13 and E17. **A** **and B**: High frequency neuron at E13 with total dendritic length of 398 *µ*m. In **B** note the dense plexus of dendritic arbors (white arrowheads) in contrast to the well-defined dendritic branches in other regions of NL (white arrowheads in **D** and **F**). **C** **and D**: Middle frequency neuron at E13 with a total dendritic length of 702 *µ*m. **E and F**: Low frequency neuron at E13 with a total dendritic length of 1254 *µ*m. **G and H**: High frequency neuron at E17 with a total dendritic length of 245 *µ*m. **I and J**: Middle frequency neuron at E17 with a total dendritic length of 578 *µ*m. Note the decrease in the number of primary dendrites from E13 to E17 (compare white arrows in **C** to **I**), particularly on the dorsal side. **K and L**: Low frequency neuron at E17 with a total dendritic length of 1154 *µ*m. While there appears to be an equal distribution of high order branches at E13 (dashed arrow in **E**), the high order branches at E17 are located near the terminals (dashed arrow in **K**), resulting in longer primary dendrites. Scale bar = 15 *µ*m A–D and G–J; 30 *µ*m E and F; 50 *µ*m K and L. Dorsal is up in all panels.

We next examined a larger set of neurons, including those filled in coronal sections, to characterize features of the tonotopic gradient of NL dendrites and to determine how they change between E13 (*n* = 66) and E17 (*n* = 43). The number of primary dendritic branches was positively correlated with total dendritic length at E13 (slope  =  + 0.004, r = 0.28, *P*<0.05; Fig. 2A, 2C, 2E, and 3A). Based on the high correlation between the total dendritic length and the tonotopic position, these results suggest that lower frequency (caudolateral) NL neurons have more primary dendrites at this age than high frequency (rostromedial). From E13 to E17 there was a 1.5-fold decrease in the slope, resulting in a significant (*P*<0.05) negative correlation at E17 (slope  =  - 0.006, r = 0.31, *P*<0.05; Fig. 2G, 2I, 2K, and 3B). A negative relationship between the number of primary dendrites and a neurons position along the tonotopic axis is consistent with previous studies that observed progressively fewer primary dendrites towards the caudolateral (low frequency) pole of NL [14], [15]. These data suggest that the gradient of primary dendrites, a significant aspect of the gradient of NL dendritic morphology, begins to emerge in NL between E13 and E17.

#### Length of Primary Dendrites

**Figure 3:**
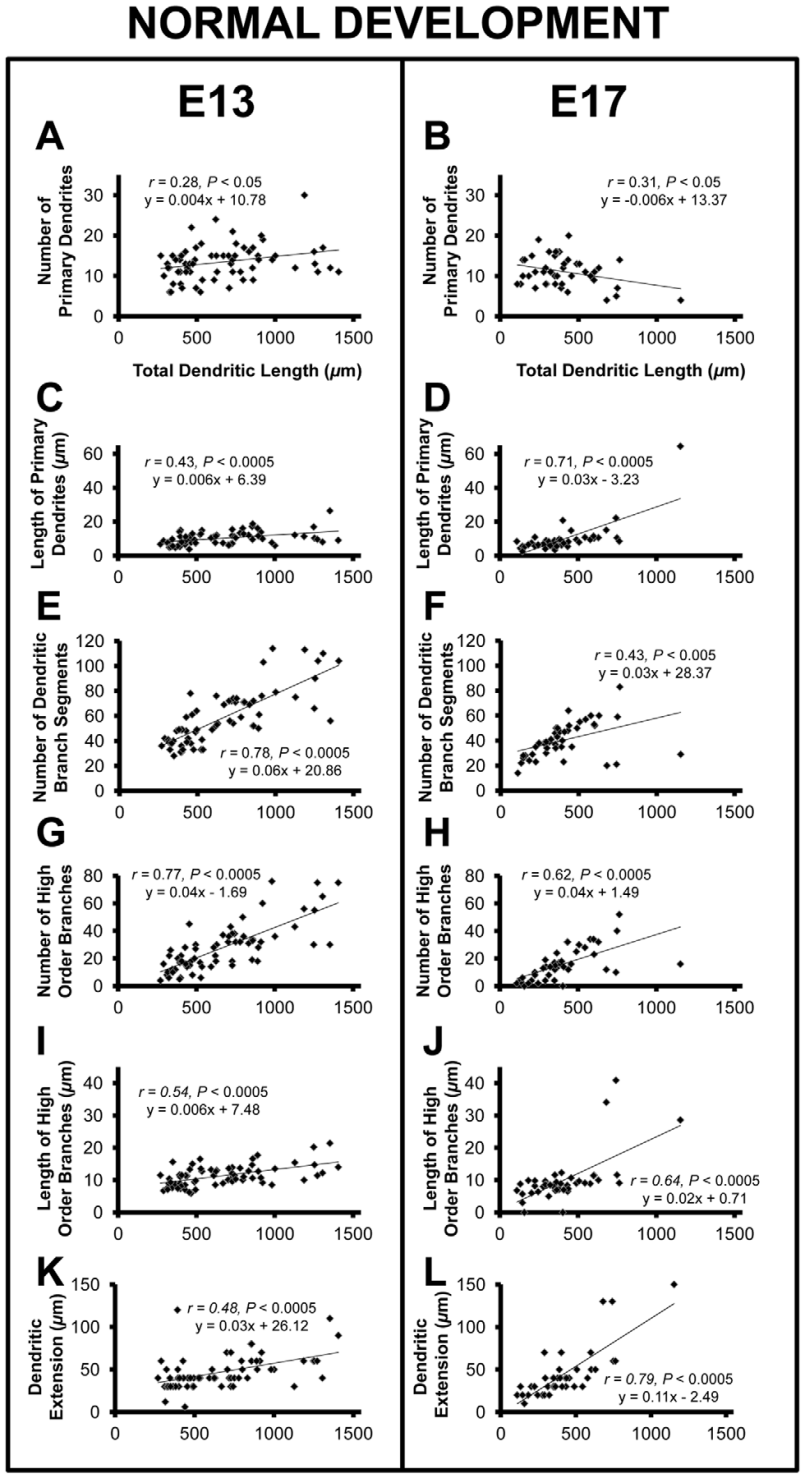
Properties of NL neurons along the tonotopic axis at E13 and E17. Neurons sampled from E13 (*n* = 66) and E17 (*n* = 43) brainstems. Scatter plot, least-squares regression, and correlation coefficient for the following features which demonstrate the correlation between: **A and B** the number of primary dendrites to total dendritic length and a significant decrease (*P*<0.0005) in the slope from E13 to E17; **C and D** the length of primary dendrites to total dendritic length and a significant increase (*P*<0.005) from E13 to E17; **E and F** the number of dendritic branch segments to total dendritic length, as well as a significant decrease (*P*<0.05) in the slope from E13 to E17; **G and H** the number of high order branches to total dendritic length; **I and J** the length of high order branches to total dendritic length and a significant increase (*P*<0.0005) in the slope from E13 to E17; **K and L** dendritic extension and total dendritic length and the significant increase in the slope (*P*<0.0005) from E13 to E17. Together the changes from E13 to E17 result in the formation of the gradient of dendritic arbor size in NL.

Consistent with previous studies demonstrating a significant relationship between position and primary dendrite length [14], we found a positive correlation between total dendritic length and length of primary dendrites that became more pronounced with age. The slope at E17 (slope  =  + 0.03, r = 0.71, *P*<0.0005; Fig. 2G, 2I, 2K, and 3D) was significantly greater (*P*<0.05) than the slope obtained at E13 (slope  =  + 0.006, r = 0.43, *P*<0.0005; Fig. 2A, 2C, 2E, and 3C).

#### Total Number of Dendritic Branch Segments

We found a positive relationship (slope  =  + 0.06) between total dendritic length and total number of dendritic branch segments at E13 (r = 0.78, *P*<0.0005; Fig. 2A–F and 3E). The number of dendritic branch segments was also positively correlated with total dendritic length at E17 (slope  =  + 0.03, r = 0.43, *P*<0.005; Fig. 2G–L, and Fig. 3F), but the slope decreased significantly from that at E13 (*P*<0.05).

#### High Order Branches

We found that the number of high order branches, including any branches of tertiary or higher order, varied systemically with neuron position, but did not change (*P* = 0.54) from E13 (slope  =  + 0.04, r = 0.77, *P*<0.0005; Fig. 3G) to E17 (slope  =  + 0.04, r = 0.62, *P*<0.0005; Fig. 3H). The length of high order branches was also positively correlated with total dendritic branch length at E13 (slope  =  + 0.006, r = 0.54, *P*<0.0005; Fig. 3I), and increased significantly (*P*<0.0005) from E13 to E17 (slope  =  + 0.03, r = 0.64, *P*<0.0005; Fig. 3J)

#### Dendritic Extension

Another critical aspect to the perceived gradient in the mature NL is the extension of dendrites from the cell body. To measure dendritic extension we performed a Sholl analysis, which represented the qualitative observation that low frequency neurons had dendrites that terminated further from the cell body than high frequency neurons [14]. At E13 we found that there was a gradual, but significant correlation between total dendritic length and dendritic extension (slope  =  + 0.03, r = 0.48, *P*<0.0005; compare Fig. 2A and 2E, Fig. 3K). The slope of dendritic extension is more robust at E17 (slope  =  + 0.12, r = 0.79, *P*<0.0005; compare 2G and 2K, Fig. 3L) and significantly greater than seen at E13 (*P*<0.0005; compare Fig. 2E and 2K).

### NL Dendritic Arbors *In Vitro*

The period between E15 and E19 represents the first phase of dendritic reorganization in NL [15]. Our data were obtained at the beginning of this period, as several features are already apparent by E17. The observation that GFAP-positive astrocytes appear at E15 prompted us to examine whether these astrocytes have a role in the maturation of the dendritic gradient. We addressed this question using cultured E13 brainstems that contained immature NL neurons and treated them with astrocyte-conditioned medium (ACM) from E16 astrocytes.

Three treatments will be discussed; control medium (as described previously), low-dose ACM (15–20 *µ*g/ml), and ACM (25–33 *µ*g/ml). We report means of percent change, and when possible, the slope of percent change for each feature across the tonotopic axis using the neurons' initial total dendritic length to estimate position along the tonotopic axis.

#### Total dendritic length

**Figure 4:**
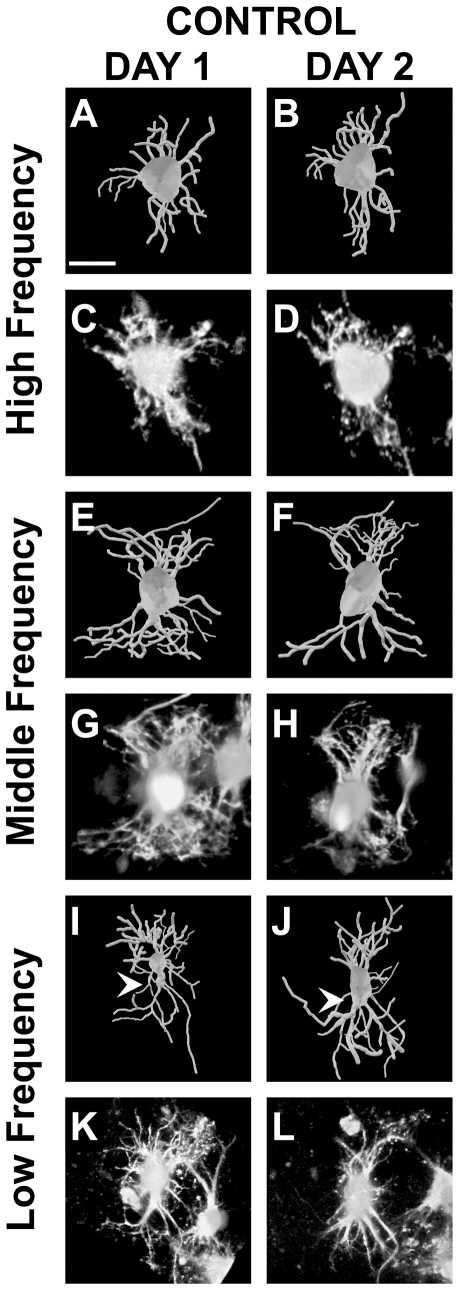
3D reconstructions and raw images of NL neurons before and after treatment with control medium. **A–D**: High frequency neuron with an initial total dendritic length of 434 *µ*m. Though there is some reorganization the overall appearance from Day 1 to 2 is unchanged. **E–H**: Middle frequency neuron with an initial total dendritic length of 901 *µ*m. The overall net change results in a small total dendritic length, but very little change in branch order distribution. **I–L**: Low frequency with an initial total dendritic length of 1132 *µ*m. There is an increase in the number primary dendrites (white arrowheads) from Day 1 to Day 2, changes opposite from what is necessary for the primary dendrite gradient at E17. Scale bars = 15 *µ*m A–H; 30 *µ*m I–L. Dorsal is up in all panels.

**Figure 5:**
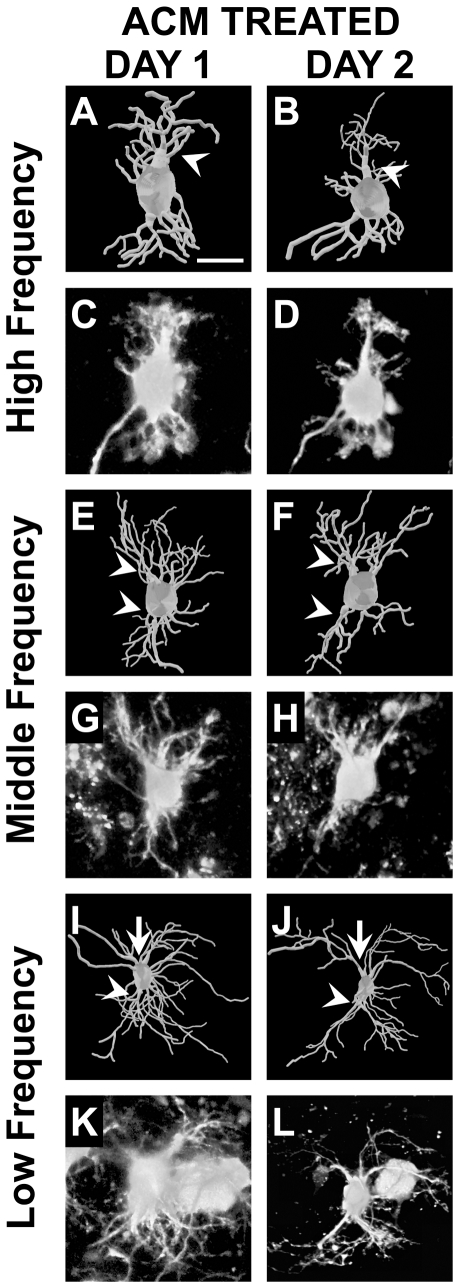
3D reconstructions and raw images of NL neurons before and after treatment with ACM. **A–D**: High frequency neuron with an initial total dendritic length of 428 *µ*m. Dorsal primary dendritic branches are reduced (−24%) to a single apically projecting branch (white arrowhead). **E–H**: Middle frequency neuron with an initial total dendritic length of 918 *µ*m. Several primary dendrites are lost (−33%), both dorsal and ventral (white arrowheads). **I–L**: Low frequency with an initial total dendritic length of 1351 *µ*m. Several primary dendrites are lost from the neuron after treatment with ACM (−61%). This effect is most visible on the ventral pole of the neuron (white arrowheads), though also apparent on the dorsal side (white arrows). The changes following treatment with ACM result in a progressive primary dendritic gradient from rostromedial to caudolateral. Scale bars = 15 *µ*m A–H, K and L; 30 *µ*m I and J. Dorsal is up in all panels.

**Figure 6:**
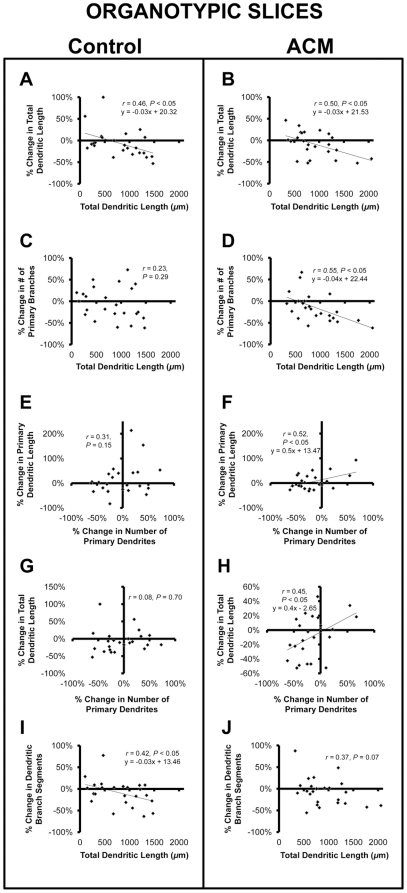
Percent change of properties of NL neurons following treatment with control medium or ACM. Neurons sampled from organotypic slice treated with control medium (*n* = 24) or ACM (*n* = 24). Scatter plot, least-squares regression, and correlation coefficient for the follow features which demonstrate: **A and B** that there is a correlation between percent change in total dendritic length and a neurons initial total dendritic length in both control and ACM treated neurons, but the slopes of percentage change are not different (*P* = 0.99); **C and D** that there is no correlation between percentage change in the number of primary dendrite number and initial total dendritic length after treatment with control medium (*P* = 0.29), but a significant negative correlation after treatment with ACM (*P*<0.05); **E and F** that there is no correlation between percent change in the number of primary dendrites and the percent change in the length of primary dendrites after treatment with control medium (*P* = 0.15), but there is a significant negative correlation (*P*<0.05) between percent change in number of primary dendrites and change in the length of primary dendrites after treatment with ACM; **H and G** that the loss of primary dendrites with ACM results in a decrease in total dendritic length (*P*<0.05), but not control medium (*P* = 0.70); **I and J** that the percent change in the number of dendritic branch segments and initial total dendritic length are significantly correlated in neurons treated with control medium (*P*<0.05), but not those treated with ACM (*P* = 0.07).

The total dendritic length decreased in neurons treated with control medium (*n* = 24, −6.41±6.7%; Fig. 4) and ACM (*n* = 24, −8.8±5.9%; Fig. 5). There was a correlation between change in total dendritic length and initial total dendritic length when treated with control medium (slope  =  −0.03, r = 0.46, *P*<0.05; Fig. 6A) and ACM (slope  =  −0.03, r = 0.50, *P*<0.05; Fig. 6B). In contrast, the total dendritic length increased in slice treated with the low-dose ACM (*n* = 21, + 9.26±10.6%), but this increase showed no correlation with initial total dendritic length (r = 0.21, *P* = 0.36; data not shown).

#### Number of Primary Dendrites

The number of primary dendrites decreased on neurons from slices treated with ACM (−15.26±6.6%), control medium (−5.13±7.7%), and low-dose ACM (−26.66±8.3%). Of the three conditions, only treatment with ACM decreased the number of primary dendrites on NL neurons as a function of their location along the tonotopic axis (slope  =  −0.04, r = 0.55, *P*<0.05; Fig. 5 and 6D). This effect results in a progressively greater reduction in the number of primary dendrites towards the low frequency region of NL. We observed no significant difference along the tonotopic axis in the change in the number of primary dendrites when the slices were treated with the control medium (r = 0.23, *P* = 0.29; Fig. 4 and Fig. 6C) or the low-dose ACM (r = 0.14, *P* = 0.55, data not shown).

We next asked to what extent the loss of primary dendrites contributed to the loss in total dendritic length. While there is no correlation between primary dendrites lost and initial primary dendrite length in neuron treated with control medium (r = 0.31, *P* = 0.15; Fig. 6E), we found a significant positive correlation in neurons treated with ACM (slope = 0.51, r = 0.52, *P*<0.05; Fig. 6F). As a result, the decrease in the number of primary dendrites in neurons treated with ACM is significantly correlated with the decrease in total dendritic length (slope = 0.40, r = 0.45, *P*<0.05; Fig. 6H). We did not observe a correlation with total dendritic length in neurons treated with control medium (r = 0.08, *P* = 0.70; Fig. 6G).

#### Length of Primary Dendrites

ACM (+ 6.51±6.5%) and control medium (+ 13.65±13.0%) increased the length of primary dendrites, while low-dose ACM led to a decrease (−6.46±14.0%). We observed no significant effect of ACM (r = 0.006, *P* = 0.81), control medium (r = 0.15, *P* = 0.49), or low-dose ACM (r = 0.14, *P* = 0.19) on the length of primary dendrites as a function of the neurons location along the tonotopic axis.

#### Total Number of Dendritic Branch Segments

We observed a decrease in the total number of dendritic segments on neurons from slices treated with ACM (−7.04±6.6%), control medium (−9.16±6.2%), and low-dose ACM (−2.7±7.0%). These effects were not different from one another (*P* = 0.79). The decrease of dendritic segments on neurons treated with the control medium (slope  =  −0.03, r = 0.42, *P*<0.05; Fig. 4 and 6I) was significantly correlated as a function of the neurons position along the tonotopic axis. We found no correlation between loss of dendritic segments and total dendritic length in neurons treated with ACM (r = 0.37, *P* = 0.07; Fig. 6J) or low-dose ACM (r = 0.07, *P* = 0.77, data not shown).

#### High Ordered Branches

Control medium decreased the number of high order branches (−10.66±9.7%), while both ACM (+ 8.7±14.1%) and the low-dose ACM result in an increase (+ 20.20±0.18%). There was no effect of ACM (r = 0.32, *P* = 0.13), control medium (r = 0.008, *P* = 0.97), or low-dose ACM (r = 0.02, *P* = 0.94) that varied as a function of tonotopic position of the neurons. There was a mean decrease in the length of high ordered branches on neurons treated with ACM (−16.24±7.5%) and control medium (−1.31±6.0%), but an increase in length on neurons treated with the low-dose ACM (17.25±0.10%). The increase with low-dose ACM was significantly different compared to ACM (*P*<0.05). There was no difference between ACM and control treatments (*P* = 0.36) or between controls and low-dose ACM (*P* = 0.23). No effect was found to be correlated with the change in length of high order branches and initial total dendritic length whether treated with ACM (r = 0.04, *P* = 0.85), control medium (r = 0.31, *P* = 0.14), or low-dose ACM (r = 0.08, *P* = 0.75).

#### Dendritic Extension

Dendritic extension varied as a function of initial total dendritic length of neurons treated with ACM (slope  =  + 0.02, r = 0.47, *P*<0.05) and the control medium (slope  =  + 0.04, r = 0.58, *P*<0.005), but this relationship was not different from the gradient at E13 (*P* = 0.44 and 0.71, respectively). There was no observable correlation between neuron location and dendritic extension in our low-dose ACM group (r = 0.20, *P* = 0.39). Treatment with ACM (r = 0.36, *P* = 0.08), control medium (r = 0.38, *P* = 0.08), or low-dose ACM (r = 0.31, *P* = 0.17) had no significant effect on percentage change in dendritic extension.

## Discussion

The goal of this study was to determine the effect of GFAP-positive astrocytes on NL dendrites during a period of extensive reorganization that results in a steep tonotopic gradient in dendritic arbor size and morphology. We established criteria to identify the tonotopic location of NL neurons filled within coronal slices. Using total dendritic length to estimate position, we examined several properties of NL neurons, first at an immature stage (E13) and then several days later (E17) after the emergence of the dendritic arbor gradient. When neurons in organotypic slices isolated from E13 brainstems were treated with ACM, we found that there was a progressive decrease in the number of primary dendrites along the tonotopic axis in such a way that recapitulated normal development. These data suggest that astrocyte-derived cues are capable of modulating the number of primary dendritic branches during development.

### Dendritic Reorganization in NL Coincides with Emergence of Astrocytes

The reorganization of NL dendrites coincides with the emergence of GFAP-positive astrocytes, which send processes into the NL neuropil at E16 [26]. The first quantitative changes in NL dendrites were previously reported between E15 and E19 [15]. Our analysis of NL neurons at E17 allowed us to compare dendritic arbors immediately after the emergence of astrocytes to relatively immature NL dendrites at E13. Consistent with previous findings, we found that total dendritic length was significantly correlated with the positions of neurons along the frequency axis at an early age [15]. At E17 there was a significant loss in the number of primary dendrites, particularly in the low frequency region of NL, resulting in a negative correlation between primary branch number and total dendritic length. Additionally, the tonotopic gradient in the length of primary dendrites was steeper at E17 than at E13, providing a key element to the mature perceived gradient [14]. There was also a reduction in the overall number of dendritic branch segments, which reflects the qualitative observation that dendritic arbors lose immature branches during the period between E14 and E16 [15].

#### Changes in the Absence of Activity

Excitatory synapses are already densely populated along NL dendrites by E10-11 [50], which roughly corresponds to the age at which auditory stimuli can evoke a response from the VIIIth nerve [51], [52]. Behavioral responses to auditory stimuli can be observed at E14 [53]. We and others [15] have observed that significant reorganization occurs between E15 and E17, several days after activity is present. Our data suggest that this reorganization is due, at least in part, to the emergence of astrocytes.

Manipulations in early embryos that prevent the formation of the ear result in the death of 30% of NM neurons [42]. Despite this loss in afferent activity, the spatial gradient is apparent in NL at E17 [18]. Without afferent activity, the possibility that sound stimulation from the cochlea drives the dendritic gradient seems unlikely [15]. It has been proposed that neurons may intrinsically regulate dendritic fields [17], and while this may be true, one key factor that had not been excluded from these preparations is the presence of astrocytes, which emerge as early at E15 and coincide with this first phase of dendritic reorganization.

### Gradient of the Number of Primary Dendrites is Modulated by Astrocyte-derived Molecules

One of most salient features of the mature dendritic gradient is the paucity of primary dendrites in the low frequency region of NL, in contrast to the extensive plexus of short branches in the high frequency region [14], [15]. We report that there is positive correlation between the number of primary dendrites and neuron location at E13, which opposes of the gradient seen at mature ages. Smith found no significant correlation at E15 along the rostromedial to caudolateral extent [15], and we report here a significant negative correlation at E17. This gradient thus emerges just after the maturation of GFAP-positive astrocytes around NL [26]. Organotypic brainstem slices from E13 embryos exposed to ACM had fewer primary dendrites, with consistently greater decreases in lower frequency neurons. These results suggest that an early function of these astrocytes is to promote maturation of primary dendrite number in NL.

While we examined several morphological features of NL neurons, the reduction in the number of primary dendrites stood out in its significant effect of ACM on NL dendrites *in vitro*. There was no correlation between change in total number of dendritic branch segments and relative position along the tonotopic axis, consistent with the possibility that the loss in primary dendrites does not account for the overall reduction in dendritic area seen during normal development. The data further suggest that individual features of dendritic morphology are independently regulated.

#### ACM Selectively Eliminates Primary Dendrites to Establish Gradient of Dendritic Arbor Size

We found that ACM significantly and progressively decreased the number of primary dendrites, but that the change in total dendritic length was not different from treatment with control medium. However further analysis can be performed to address this apparent contradiction. Since neurons in the caudolateral portion of NL are initially much longer than those in the rostromedial portion [15], pruning of longer than average primary dendrites could generate and maintain a constant slope of total dendritic length across the tonotopic axis. Consistent with this possibility, we found that percent change in the length of primary dendrites correlated with the percent change in the number of primary dendrites with ACM treatment. To extend our findings further, the percentage of primary dendrites lost after ACM treatment was significantly correlated with a decrease in total dendritic length. Thus we conclude that the decrease in total dendritic length can be modulated by reducing the number of primary dendrites, which is modulated by astrocyte-secreted molecules.

Despite the loss of primary dendrites, there was no apparent redistribution of dendritic area into permanent branches since change in dendritic extension was also absent. High ordered branches become significantly longer at E17, but we did not observe this latter phase *in vitro*. It is possible that additional growth and the increase in dendritic extension occurs following afferent stimulation and hearing onset [20], [25], [54]. Taken together, ACM selectively eliminates primary dendrites and may affect other morphological properties of NL neurons.

### Potential Mechanism that Modulate Primary Dendrite Number

In this study we showed that brainstem astrocyte-derived molecules can modulate the number of primary dendrites along the tonotopic axis during development. Our findings on primary dendrites of aspiny neurons may be distinct from astrocyte control in other systems. Dendrites are reported to interact in direct contact with glia during the formation of dendritic spines via Eph receptor tyrosine kinases [55], [56]. We have shown that some of the Eph proteins are localized to NL dendrites and show tonotopic expression gradients in NL neurons and surrounding astrocytes [36], [57]. Because these receptors bind to membrane-associated ligands [58], [59], [60], it is unlikely that the factors secreted from astrocytes act directly through these receptors, but it remains possible that contact-mediated signaling from astrocytes provides additional regulation of NL neurons through these proteins. Other molecular interactions, elicited by secreted molecules, must thus be considered.

#### Developmental Regulation of Dendrites

Molecular mechanisms that contribute to dendritic development in the auditory brainstem have not been identified. However, a similar process of dendritic elaboration can be found in the cerebellum, a region of relatively similar embryonic origin [61]. As with NL dendrites in the absence of VIIIth nerve afferents [17], [18], [42], Purkinje cells are capable of forming aspects of their characteristic dendritic arbor *in vitro* without glutamatergic activity [62]. Purkinje cells in the cerebellum grow along Bergmann glia, where signaling between the two has been shown to be necessary for arbor elaboration [33], [63]. Similar to the changes that occur in the low frequency neurons in NL, Purkinje cells lose a significant number of their primary dendrites during development [64]. Several intrinsic and extrinsic molecular interactions have been shown to be involved in regulating the formation of these dendritic arbors [65] including neurotrophins [66] as well as glial interactions through pathways yet unknown [67].

Neurotrophins are known to regulate neuron differentiation and dendritic morphology through the tropomyosin receptor kinases (Trks) [68]. TrkB and TrkC are expressed in NL throughout the NL neuropil during development [69], and TrkB is localized to the ventral neuropil after about E10 [57], [69], but does not appear to be expressed in a tonotopic gradient. TrkC has been found to be developmentally regulated in the mammalian ventral cochlear nucleus [70] and its ligand, NT3 can be localized along astrocytic processes in the rat cochlear nucleus [71]. Whether neurotrophins regulate primary dendrites via neuron-glia interactions remains to be determined.

#### Tonotopically Ordered Molecules in NL Neurons

In our studies, bath application of ACM results in a progressively more robust effect on neurons in the caudolateral region of NL than those in the rostromedial part of the nucleus. We have not observed a gradient of astrocyte cell bodies or processes along the tonotopic axis of NL [26], suggesting that the release of factors from astrocytes may be homogenous throughout the neuropil. Since we observed a differential effect of the bath-applied astrocyte-derived molecules in our preparation, it is likely that binding partners for ACM-derived signals have graded expression or function in NL neurons.

While the identity of these molecules is not known, several proteins show tonotopically graded expression in NL. Receptors such as the Kv1.3 potassium channel, necessary for neurons that process high frequency stimulation [72], are developmentally regulated [28] and are expressed in a tonotopic gradient [73]. Changes in the expression of AMPA-type glutamate receptors coincide with several morphological changes during the development of the owl auditory brainstem [74], [75], [76], and may mediate signals involved in dendritic morphogenesis. The NMDA receptor subunit 1 is developmentally regulated and is expressed tonotopically throughout the NL cell body layer and neuropil by E14 [77], just prior to the emergence of astrocytes. Receptor stimulation can promote calcium signaling [78], which then opens up several possible intrinsic mechanism for affecting dendritic morphology (reviewed in [65], and [79]).

### Dendritic Morphology in Diseases of the Central Nervous System

The experiments described here make for a promising avenue of research that explores the role of astrocytes in development of the auditory brainstem, but the observation that astrocyte affect neuronal morphology has wide reaching importance in the field of neuronal dysfunction and disease. Compelling evidence comes from studies in mouse models of Rett syndrome [80] and fragile X syndrome [81], both neurodevelopmental disorders associated with mental retardation, seizures, and autistic behaviors. Astrocytes from both types of mutant mouse produced abnormal dendritic morphology in wild type hippocampal neurons. Conversely, mutant hippocampal dendritic morphology was rescued by co-culture with wild type astrocytes. There is also evidence from studies on fetal alcohol syndrome that suggests astrocytes can prevent damage of ethanol-induced dendritic atrophy [82].

These observations suggest that astrocytes shape dendritic morphology during normal development, and that failure of this regulation is associated with severe cognitive and behavioral symptoms. While these studies only investigated hippocampal neurons, dysregulation of dendrites in other brain areas may contribute to these symptoms. In autistic patients, who often have hearing problems, morphology and orientation is disrupted in neurons in the middle superior olive (MSO) [83], which is analogous to NL. These finding are not exclusive to MSO and extend to several other auditory nuclei [84]. Irregular morphology results in disrupted tonotopic organization in a rat model of autism, suggesting that a common defect is responsible for these malformations [85]. These studies highlight the importance of dendritic morphology in CNS function and underscore the importance of our findings on the role of astrocytes in dendritic development.
